# Supplementary material for: Encapsulation of Hemp (Cannabis sativa L.) Essential Oils into Nanoemulsions for Potential Therapeutic Applications: Assessment of Cytotoxicological Profiles
Source: Molecules. 2023 Sep 7;28(18):6479. doi: 10.3390/molecules28186479 (PMC10537312; doi:10.3390/molecules28186479)
Supplement: Supplementary file 1 [file molecules-28-06479-s001.zip › molecules-2550108-supplementary.pdf]

molecules

Supplementary materials

Encapsulation of hemp (*Cannabis sativa* L.) essential oils into nanoemulsions for potential therapeutic applications: assessment of cytotoxicological profile

Cristina Aguzzi[1#], Diego Romano Perinelli[2#], Marco Cespi[2], Laura Zeppa[1], Eugenia Mazzara[2], Filippo Maggi[2], Riccardo Petrelli[2], Giulia Bonacucina[2*], Massimo Nabissi[1*]

[1] *Department of Experimental Medicine, School of Pharmacy, University of Camerino, Via Madonna delle Carceri 9, 62032, Camerino, Italy*
[2] *Chemistry Interdisciplinary Project (ChIP) Research Center, School of Pharmacy, University of Camerino, Via Madonna delle Carceri, 62032, Camerino, Italy*

* Correspondence: giulia.bonacucina@unicam.it; massimo.nabissi@unicam.it
[#] These authors equally contributed

Table S1. Complete GC-MS characterization of Uso 31 and Carmagnola CS EOs.

N	Component[a]	RI Calc.[b]	RI lit.[c]	% Uso 31	% Carmagnola CS
1	α-Thujene	921	924	0.02	0.02
2	α-Pinene	926	932	11.07	13.45
3	Camphene	939	946	0.04	0.17
4	Sabinene	966	967	0.04	
5	β-Pinene	968	974	3.59	5.45
6	Myrcene	989	988	15.28	37.57
7	α-Phellandrene	1003	1002	0.13	0.08
8	δ-3-Carene	1008	1008	0.06	0.09
9	α-Terpinene	1014	1014	0.02	0.07
10	Limonene	1025	1024	1.59	5.37
11	(1,8)-Cineole	1027	1026		0.43
12	(*Z*)-β-Ocimene	1037	1032	0.21	0.16
13	(*E*)-β-Ocimene	1047	1044	6.71	1.79
14	γ-Terpinene	1056	1054	0.02	0.08
15	Terpinolene	1085	1086	5.46	2.97
16	(*Z*)-Caryophyllene	1407	1408		0.23
17	(*E*)-Caryophyllene	1409	1417	25.93	16.99
18	α-*trans*-Bergamotene	1430	1432	2.13	0.19
19	α-Humulene	1443	1452	8.92	6.11
20	*allo*-Aromadendrene	1450	1458	1.32	0.18
21	(*E*)-β-Farnesene	1456	1454	1.70	0.06
22	β-Selinene	1476	1489	3.35	1.16
23	α-Selinene	1485	1498	1.96	0.89
24	α-Bulnesene	1507	1509		0.22
25	δ-Cadinene	1519	1522		0.04
26	Selina-3,7(11)-diene	1531	1545		0.11
27	Caryophyllene oxide	1571	1582	7.23	2.95
28	Humulene epoxide II	1593	1608	1.81	0.63
29	Cannabidiol	2419	2430	0.43	0.23
	**Total identified (%)**			99.03	97.72
	Monoterpene hydrocarbons (%)			44.24	67.27
	Oxygenated monoterpenes (%)				0.43
	Sesquiterpene hydrocarbons (%)			45.31	26.18
	Oxygenated sesquiterpenes (%)			9.04	3.58
	Cannabinoids (%)			0.43	0.23

Disclaimer/Publisher's Note: The statements, opinions and data contained in all publications are solely those of the individual author(s) and contributor(s) and not of MDPI and/or the editor(s). MDPI and/or the editor(s) disclaim responsibility for any injury to people or property resulting from any ideas, methods, instructions or products referred to in the content.
